# Wordline Input Bias Scheme for Neural Network Implementation in 3D-NAND Flash

**DOI:** 10.3390/biomimetics10050318

**Published:** 2025-05-15

**Authors:** Hwiho Hwang, Gyeonghae Kim, Dayeon Yu, Hyungjin Kim

**Affiliations:** Division of Materials Science and Engineering and Department of Semiconductor Engineering, Hanyang University, Seoul 04763, Republic of Korea

**Keywords:** computing-in-memory (CIM), 3D-NAND architecture, neuromorphic computing, vector-matrix multiplication (VMM), velocity saturation

## Abstract

In this study, we propose a neuromorphic computing system based on a 3D-NAND flash architecture that utilizes analog input voltages applied through wordlines (WLs). The approach leverages the velocity saturation effect in short-channel MOSFETs, which enables a linear increase in drain current with respect to gate voltage in the saturation region. A NAND flash array with a TANOS (TiN/Al_2_O_3_/Si_3_N_4_/SiO_2_/poly-Si) gate stack was fabricated, and its electrical and reliability characteristics were evaluated. Output characteristics of short-channel (*L* = 1 µm) and long-channel (*L* = 50 µm) devices were compared, confirming the linear behavior of short-channel devices due to velocity saturation. In the proposed system, analog WL voltages serve as inputs, and the summed bitline (BL) currents represent the outputs. Each synaptic weight is implemented using two paired devices, and each WL layer corresponds to a fully connected (FC) layer, enabling efficient vector-matrix multiplication (VMM). MNIST pattern recognition is conducted, demonstrated only a 0.32% accuracy drop for the short-channel device compared to the ideal linear case, and 0.95% degradation under 0.5 V threshold variation, while maintaining robustness. These results highlight the strong potential of 3D-NAND flash memory, which offers high integration density and technological maturity, for neuromorphic computing applications.

## 1. Introduction

Artificial intelligence (AI) has advanced rapidly in recent years, driven by improvements in machine learning algorithms, increased computational power, and the availability of large datasets. However, conventional computing systems based on the von Neumann architecture suffer from inherent limitations due to the physical separation of memory and processing units [1]. This limitation, known as the von Neumann bottleneck, poses major challenges for real-time processing of large-scale data, particularly under the increasing demands of AI and edge computing. As conventional von Neumann computing architectures face fundamental limitations in energy efficiency and latency, neuromorphic computing, inspired by biological nervous systems, is increasingly considered a promising alternative. To overcome these limitations, computing-in-memory (CIM) has emerged as a promising solution. Unlike traditional architectures, CIM integrates computation within memory arrays, significantly alleviating data transfer bottlenecks and improving energy efficiency. This paradigm is especially well-suited for accelerating deep neural network (DNN) workloads [2,3,4,5,6,7,8,9,10,11,12].

DNNs, which drive advancements in AI, require highly efficient computational architectures. CIM has been recognized as a promising approach for accelerating core operations, such as vector-matrix multiplication (VMM), which are central to DNN computations. Various non-volatile memory devices, including resistive random-access memory (ReRAM) [13,14,15,16,17,18,19,20,21,22,23,24,25,26], phase-change memory (PCM) [27,28,29,30,31,32,33,34,35,36], and ferroelectric devices [37,38,39,40,41,42,43,44,45,46], have been investigated for CIM applications due to their distinctive properties. Among them, flash memory has gained significant attention in the CIM application [47,48,49,50,51,52,53,54,55,56]. In particular, NAND flash stands out as a highly attractive candidate owing to its high memory density, technological maturity, and excellent scalability. Compared to other emerging memory devices, its advanced development and manufacturing readiness make it especially suitable for the increasing demands of AI and edge computing systems.

However, 3D-NAND flash presents unique challenges, particularly in supporting analog input methodologies. The nonlinear current–voltage (*I*–*V*) characteristics of field-effect transistors (FETs), combined with the vertically stacked architecture, complicate the processing of analog data inputs. Although several studies have explored binary or ternary input schemes via wordlines (WLs) [57], these approaches often suffer from accuracy degradation due to data loss associated with non-analog encoding. In contrast, analog input can minimize data loss and ensure a linear relationship between input and output, which is critical for fully connected (FC) layers. Nonetheless, implementing analog input remains difficult because of the quadratic dependence of drain current on gate voltage in a transistor. To address this issue, alternative methods such as bitline (BL)-based input and pulse-width modulation (PWM) techniques have been proposed; however, the implementation of multi-level inputs and weights in these approaches relies on shift-and-adder circuits, along with a large number of cells and BLs, leading to reduced area efficiency and cell density [58,59,60]. Additionally, analog inputs implemented using PWM introduce inference latency, which depends on the input values, while also requiring additional peripheral circuitry for input encoding. Lastly, analog input through BLs operating in the device linear region inherently limits the usable voltage range, thereby constraining the flexibility of analog signal representation.

In this work, we present a method for applying analog input through WLs while maintaining the conventional input/output terminal configuration of the 3D-NAND flash architecture and preserving analog data processing. This approach utilizes the velocity saturation effect observed in short-channel devices, where under high drain voltage conditions, the electric field between the source and drain becomes sufficiently strong to saturate carrier velocity. Consequently, the drain current exhibits a near-linear dependence on gate voltage, unlike the quadratic behavior observed in long-channel devices. By exploiting this characteristic, we can achieve the representation of linear analog input through the WLs, with a relatively wider range compared to the BL-based scheme. Also, by utilizing two distinct memory states, program (PGM) and erase (ERS), within a wide memory window that provides a large difference in current, the proposed scheme exhibits strong tolerance to threshold voltage variations. A charge trap-flash (CTF)-based NAND array was fabricated using a TANOS (TiN/Al_2_O_3_/Si_3_N_4_/SiO_2_/poly-Si) flash stack, and its electrical and memory characteristics were experimentally evaluated. Transfer characteristics were compared across devices with different gate lengths, confirming the linear response of short-channel devices. The current-voltage characteristics were applied to a neural network, and the system performance was analyzed against an ideal software-based linear input function. In addition, the robustness of the system was evaluated under threshold voltage variation in the PGM state, demonstrating its suitability for analog neural computing.

## 2. Device Fabrication and Electrical Characteristics

**Figure 1:**
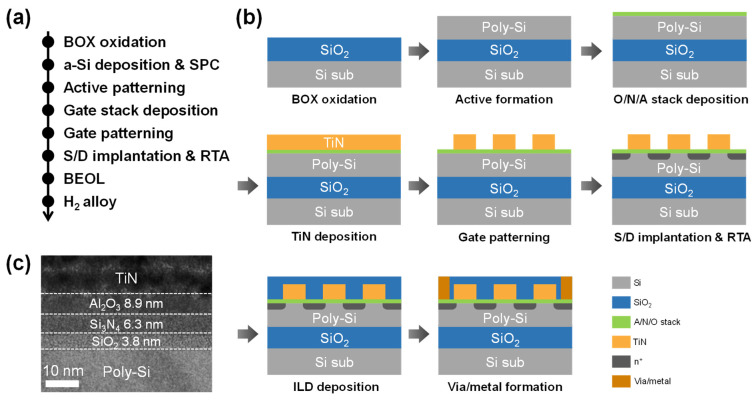
Fabrication of NAND flash array with TANOS stack. (**a**) Process flow of the fabricated NAND flash device. (**b**) Schematic diagram for each process step. (**c**) Cross-sectional TEM image of TANOS gate stack.

Figure 1a shows the process flow of the fabricated NAND flash array, while Figure 1b presents schematic illustrations of each corresponding process step. Initially, a 300 nm-thick buried oxide layer was formed through wet oxidation on a p-type bulk silicon wafer. Then, 100 nm-thick amorphous silicon was deposited at 550 °C using low-pressure chemical vapor deposition (LPCVD). Subsequently, solid-phase crystallization (SPC) was carried out by annealing at 600 °C for 24 h, resulting in the formation of a 100 nm thick poly-Si channel layer. Active area patterning and isolation were then performed through dry etching. Then, 3.8 nm-thick SiO_2_ tunneling oxide and a 6.3 nm-thick Si_3_N_4_ charge trap layer were deposited by LPCVD, followed by the deposition of an 8.9 nm-thick Al_2_O_3_ blocking oxide using the atomic layer deposition (ALD). A 50 nm TiN gate electrode was then deposited using metal-organic chemical vapor deposition (MOCVD), followed by the addition of a 25 nm SiO_2_ hard mask through plasma-enhanced chemical vapor deposition (PECVD) to enhance adhesion between the TiN gate and the photoresist. Gate patterning was followed by etching processes, where the hard mask region was etched using buffered oxide etchant. Subsequently, self-aligned source/drain ion implantation was carried out using As⁺ ions at 40 keV with a dose of 2 × 10^15^ cm^−2^, followed by dopant activation via rapid thermal annealing (RTA) at 950 °C for 10 s. Afterward, the BEOL process, including inter-layer dielectric (ILD) deposition, via hole formation, and metallization followed. Finally, forming gas annealing (FGA) was performed at 450 °C for 30 min. Figure 1c shows a cross-sectional transmission electron microscopy (TEM) image of the TANOS gate stack in the fabricated flash device, demonstrating proper layer formation.

**Figure 2:**
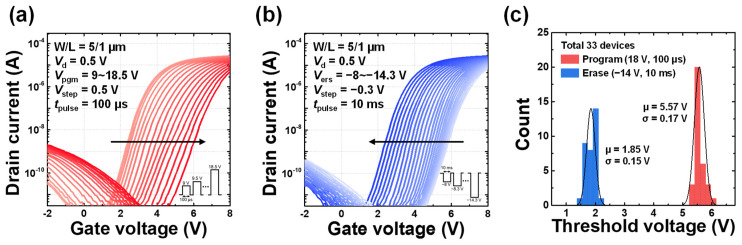
Electrical characteristics of the fabricated device. (**a**) Measured *I*_d_-*V*_g_ transfer characteristics with ISPP scheme. (**b**) Measured *I*_d_-*V*_g_ transfer characteristics with ISPE scheme. (**c**) Threshold voltage distributions of PGM and ERS states measured from 33 devices.

The electrical properties of the fabricated device were characterized with a channel width (*W*) of 5 μm and gate length (*L*) of 1 μm. The electrical measurements were performed using a Keysight B1500A semiconductor parameter analyzer. The transfer characteristics (*I*_d_-*V*_g_) were obtained using the source measure unit (SMU), while write operations of PGM and ERS pulses were applied through the pulse generator unit (PGU). To evaluate the memory characteristics of the fabricated device, incremental step pulse program (ISPP) and incremental step pulse erase (ISPE) methods were employed. Figure 2a presents the measured transfer curves under ISPP operation. For program operation, program voltage (*V*_pgm_) was increased in 0.5 V steps from 9 V to 18.5 V with a pulse width (*t*_pulse_) of 100 µs. Conversely, Figure 2b shows the transfer curves under the ISPE operation, where erase voltage (*V*_ers_) was decreased in −0.3 V steps from −8 V to −14.3 V with a pulse width of 10 ms. In both operations, the drain voltage (*V*_d_) was set to 0.5 V. Figure 2c shows the device-to-device threshold voltage distributions for the PGM and ERS states measured from 33 devices. For each state, a *V*_pgm_ of 18 V with a width of 100 μs and a *V*_ers_ of −14 V with a width of 10 ms were applied. This confirms that both states follow a Gaussian distribution well.

**Figure 3:**
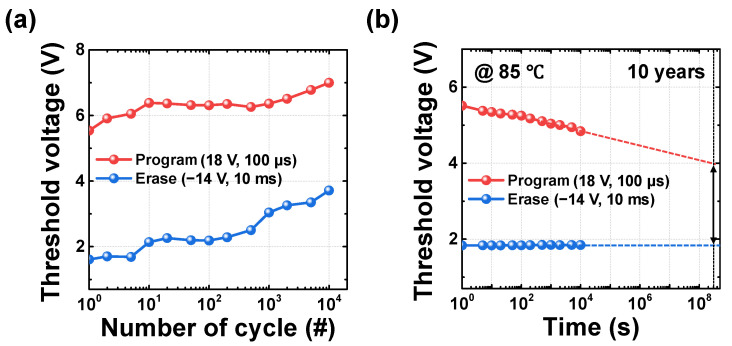
Memory characteristics. (**a**) Endurance characteristics for 10^4^ cycles. (**b**) Retention characteristics measured at 85 °C.

Figure 3a presents the endurance characteristics over 10^4^ PGM and ERS cycles. A *V*_pgm_ of 18 V with a width of 100 μs and a *V*_ers_ of −14 V with a width of 10 ms were applied, under the same conditions used in Figure 2c. Even after 10^4^ cycles, the device maintains a memory window of approximately 3.28 V, indicating robust endurance performance. Figure 3b illustrates the retention characteristics of the programmed and erased states, evaluated at a temperature of 85 °C. Similar to the endurance measurement, the device was programmed with a *V*_pgm_ of 18 V with a pulse width of 100 μs and erased with a *V*_ers_ of −14 V with a pulse width of 10 ms. The threshold voltage was then measured over time by repeated read operations. After 10^4^ seconds, the threshold voltage of the programmed state decreased from 5.51 V to 4.84 V (a shift of 0.67 V), while the erased state showed shifting from 1.83 V to 1.84 V (a shift of 0.01 V). Extrapolation of the curves using a log-scale plot indicates that the memory window remains greater than 2 V even after 10 years, demonstrating sufficient separation between the two states. These results confirm that the fabricated device exhibits excellent and reliable switching characteristics thanks to the mature technology of flash stack.

## 3. Wordline Input Bias Scheme for Neural Network Implementation

**Figure 4:**
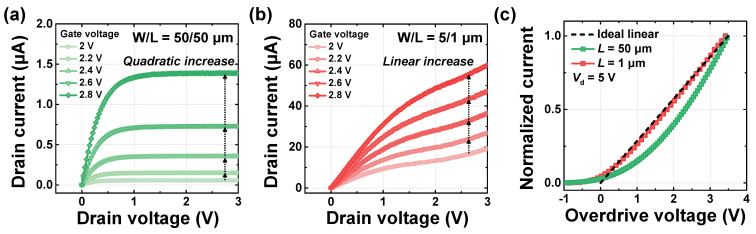
Output characteristics with varying the gate voltage (**a**) for long-channel device (*W*/*L* = 50/50 μm) and (**b**) short-channel device (*W*/*L* = 5/1 μm). (**c**) Comparison of the normalized currents of the long-channel and short-channel devices with an ideal linear function, with respect to overdrive voltage.

Figure 4a,b show the output characteristics of the flash memory devices with *L* of 50 μm and 1 μm, respectively. This confirms that the increment in drain current with respect to gate voltage becomes linear as velocity saturation occurs because of velocity saturation, for the short channel device. Theoretically, carrier velocity can be expressed as the product of mobility and the electric field applied across the channel. Under these conditions, the resulting drain saturation current is typically approximated as a quadratic function of gate voltage. However, when the electric field increases beyond a specific range, carrier velocity saturates, behaving more like a constant rather than varying with the electric field. This phenomenon causes the relationship between drain current and gate voltage to deviate from a quadratic trend and instead follow a linear relationship. Consequently, the drain saturation current can be approximated as a first-order function of gate voltage. Figure 4c shows the normalized drain current as a function of overdrive voltage (*V*_OV_), which is defined as the gate voltage with the threshold voltage subtracted, for both long-channel and short-channel devices, compared against an ideal linear function. To utilize the saturation current, the drain voltage was set to 5 V. As discussed earlier, the long-channel device exhibits a quadratic increase in current, whereas the short-channel device shows a linear increase due to velocity saturation, significantly influencing its response to varying gate voltages. To model this behavior, the region where gate voltage ranged from 0 V to 3.5 V is fitted using a third-order polynomial function for the simulation of 3D-NAND flash array to implement a deep neural network with the WL input bias scheme. This confirms that analog input data can be encoded according to the gate voltage in short-channel devices due to its linear relationship.

**Figure 5:**
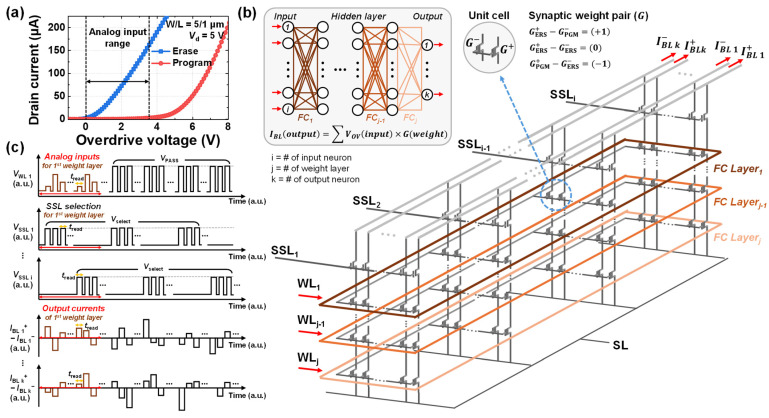
(**a**) *I*_d_-*V*_OV_ characteristics for both program and erase states, along with the corresponding analog input voltage range. (**b**) Schematic view of a 3D-NAND flash array implementing an artificial neural network with an analog WL input bias scheme. (**c**) Operation pulse diagram of WL_1_, SSLs, and BLs over time.

Figure 5a shows the *I*_d_-*V*_OV_ characteristics of both the program and erase states of the short-channel device for neuromorphic computing applications. Within the memory window defined by the two states, the erase state exhibits a linear increase in drain current with respect to the gate voltage, which is attributed to velocity saturation. This characteristic enables the use of the memory window region for analog input voltage range. In addition, the device in the erase state operates in the on-current regime, while the program state remains in the subthreshold region, thereby ensuring a large current difference between the two states. This linear *I*–*V* relationship enables the application of input data as *V*_OV_ input voltages through the WLs in the 3D-NAND array.

Figure 5b presents the schematic view of the WL input biasing scheme based on the 3D-NAND flash architecture, incorporating the synaptic weight layer. The proposed neuromorphic system performs VMM operations using a weight summation approach based on the following input and output weight configurations: $I_{BL}\left(output\right)=\sum V_{OV}\left(input\right)\;\times \;G(weight)$*,* where *I_BL_* represents the BL current, *V_OV_* is the input analog WL voltage, and *G* represents the synaptic weight stored in each cell. Each unit cell consists of two devices, one storing a positive weight value (*G*^+^) and the other storing a negative weight value (*G*^−^). The synaptic weight pair follows the relation ${G=G^{+}-G}^{-}$, where each weight cell is programmed or erased to represent ternary weight states (−1, 0, 1). This configuration allows a single pair of cells to store a weight equivalent to that in the software. Additionally, each FC layer is implemented as a dedicated WL layer, where its corresponding weights of each FC layer are stored. During VMM operation, analog WL voltages are applied to the selected WL layer associated with the active FC layer, while a pass voltage (*V*_PASS_) is applied to the remaining WLs to minimize string IR drop and to ensure that computations occur only within the selected layer. The output is then obtained by summing the currents flowing through the BLs. Lastly, the number of SSLs (*i*) corresponds to the number of input neurons in the input layer, the number of WLs (*j*) corresponds to the number of FC layers in the neural network model, and the number of paired BLs (*k*) corresponds to the number of output neurons in the output layer.

Figure 5c illustrates the VMM operation scheme, presenting the pulse diagrams of the applied voltages on the WL_1_ and SSLs, along with the corresponding BL current responses over time. To obtain the desired product of input values and weights on the BL, only one SSL is activated at a time. This exclusive select operation, which reads the current from a single synaptic pair cell at a time, introduces a drawback in inference latency due to its sequential operation. However, it alleviates the issue of large parasitic capacitance typically found in conventional 3D-NAND flash read operations, which arises from the simultaneous activation of multiple SSLs. In addition, by avoiding the overlap of multiple cell currents, it effectively prevents the accumulation of discrete random telegraph noise-induced fluctuations.

During the first phase of the operation, a select voltage (*V*_select_) is applied to SSL_1_ to activate the string select transistor, the first input value *X*_1_, encoded as an analog overdrive voltage, is applied to WL_1_, and the remaining WLs (WL_2_ to WL_j_) are biased with *V*_PASS_ to minimize IR drop and avoid interference in the unselected WL layers. All of these operations are performed simultaneously during a single read pulse (*t*_read_). As a result, the corresponding BL pairs generate an output current, representing the product of the input voltage and stored weight. For instance, the sum of the currents from BL_1_^+^ and BL_1_^−^ corresponds to *X*_1_ × *W*_11_, while the sum of BL_k_^+^ and BL_k_^−^ corresponds to *X*_1_ × *W*_1k_. In the second phase, SSL_2_ is activated by applying *V*_select_, and WL_1_ is biased with the second input value *X*_2_, also encoded as voltage, while the remaining WLs continue to bias *V*_PASS_. Similar to the first phase, the BLs output currents corresponding to the product of the second input and the weights connected to the second input neuron. Specifically, BL_k_^+^ and BL_k_^−^ output *X*_2_ × *W*_k_. This process continues iteratively for all n inputs, from SSL_1_ to SSL_i_. In this example, a single-layer neural network is considered for demonstration purposes, and all operations are performed on a single WL layer. In the case of multi-layer neural networks, each FC layer is mapped to a corresponding WL layer, and read operations are performed sequentially from the input WL layer to the output WL layer.

**Figure 6:**
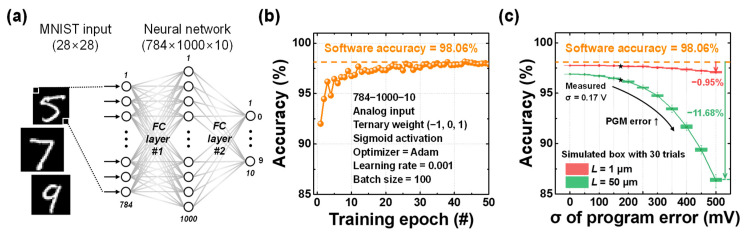
(**a**) Schematic of a neural network for MNIST pattern recognition, consisting of two FC layers with sigmoid activation functions. (**b**) Software training results using analog input, ternary weights, and sigmoid activation functions. (**c**) Thirty-trial box plot of training results utilizing the analog *I*_d_-*V*_OV_ characteristics of short-channel and long-channel devices along with the program error.

A simulation of the MNIST classification task was conducted using a neural network with a 784 × 1000 × 10 architecture, consisting of two FC layers, as illustrated in Figure 6a. The sigmoid function was used as the activation function, and the weights were quantized to ternary values (−1, 0, 1). Quantization-aware training was employed to incorporate weight quantization effects during the training process. The model was trained using the CrossEntropyLoss function and the Adam optimizer with a learning rate of 0.001. A total of 60,000 images were used for training and 20,000 for testing, with training performed over 35 epochs. Figure 6b shows the test accuracy after each epoch using an ideal linear input function, with the accuracy reaching 98.06% at the final epoch. Subsequently, simulations were performed using the fitted *I*_d_-*V*_OV_ transfer curves of the short-channel (*L* = 1 μm) and long-channel (*L* = 50 μm) devices, respectively. For both cases, variations in the threshold voltage of the PGM state were applied using a Gaussian distribution with a mean of 0 V and standard deviations ranging from 0 V to 500 mV in 50 mV increments. As shown in Figure 2c, the measured device-to-device threshold variation for the PGM state exhibited a standard deviation of approximately 170 mV. Based on this result, the simulations reflected not only the experimentally extracted variation but also larger potential errors to examine the robustness of the scheme under more severe conditions.

Figure 6c shows the recognition accuracy results, presented as box plots from 30 repeated trials for each case. First, in the case without variation in the PGM state, the recognition accuracy dropped by 1.16% for the long-channel device and by 0.32% for the short-channel device, compared to the ideal linear input function. The relatively larger accuracy drop observed in the long-channel device is attributed to the quadratic characteristic of its *I*–*V* characteristics. In contrast, although the short-channel device maintained high accuracy, a small drop was still observed. This accuracy drop is attributed to its imperfect linearity compared to the ideal function, as well as the accumulation of residual current due to its inability to fully suppress the current near the zero-input voltage point. Additionally, as the threshold voltage variation of the PGM state increases, the recognition accuracy of the long channel device significantly decreases, by up to 11.68%. This degradation is attributed to the increased shift of the PGM state toward the turn-on region within the memory window between the ERS and PGM states. In this region, although the PGM state is ideally expected to produce zero current, it begins to generate small but non-zero currents relative to the ERS state. As the threshold variation increases, the number of such undesired outputs increases, and the accumulated current eventually affects the overall output, leading to degraded recognition performance. Consequently, the long channel device exhibits a noticeable drop in accuracy. In contrast, despite the presence of such non-idealities, the short channel device shows less than a 1% accuracy drop even under a standard deviation error of 500 mV. This robustness is attributed to its near-perfect linear analog input-output characteristics, which maintain a large current difference between the PGM and ERS states even as program variation increases. As a result, the short-channel device demonstrates strong tolerance to variation and offers the advantage of achieving high accuracy by leveraging analog input behavior. 

In addition, in the current implementation, nearly the entire memory window between the PGM and ERS states is used as the input voltage range. However, if the input voltage range is limited to a narrower region within the memory window where the current difference between the PGM and ERS states is very large, the resulting accuracy drop can be reduced. This approach would not only enhance robustness against threshold voltage variation but also improve resilience to other noise sources such as RTN. Moreover, the analog WL input scheme can be effectively applied to short channel devices with channel lengths below approximately 5 μm, as long as they exhibit sufficiently linear *I*–*V* characteristics, and its effectiveness improves as the channel length decreases due to the increasingly linear behavior [61,62]. In particular, commercially available 3D vertical NAND (VNAND) devices have already been scaled down to below sub-micrometer channel lengths, and even more linear *I*–*V* characteristics can be achieved compared to the 1 μm channel devices demonstrated in this work [63]. Consequently, such commercialized VNAND is expected to maintain high recognition accuracy and exhibit enhanced robustness against variation. Furthermore, since this approach operates in the saturation region, the drain current exhibits minimal sensitivity to variations in the drain voltage. This characteristic provides tolerance in the output current with respect to fluctuations in the applied drain voltage.

## 4. Conclusions

We proposed a neuromorphic system based on 3D-NAND flash architecture that utilizes analog input voltages applied through WLs and corresponding BL current outputs. This approach utilized the velocity saturation effect in short-channel MOSFETs in the saturation region, which leads to a near-linear increase in drain current with respect to gate voltage. A NAND flash array utilizing the TANOS gate stack was fabricated, and its electrical characteristics and memory characteristics were evaluated. The output characteristics were experimentally verified to vary with the device channel length. As the channel length decreased, the drain current shifted from a quadratic to a more linear increase with respect to the gate voltage, due to the velocity saturation. By utilizing the linear transfer characteristics, the *V*_OV_ was used as the input voltage applied through the WLs. Two paired devices represented a single synaptic weight, and each WL layer corresponds to FC layer. The resulting summed BL currents represent the output values. Based on this configuration, we presented a neural network implementation using 3D-NAND flash architecture, along with the voltage biasing scheme required for VMM operations. This methodology overcame the limitations of previous studies, such as the need for additional peripheral circuits to implement multi-bit or analog input signals as well as multi-bit weights and enables the representation of a wider range of analog values with greater flexibility compared to the analog voltages through BLs. Finally, a simulation of MNIST pattern recognition was performed based on the proposed neuromorphic system. The recognition accuracy was evaluated by comparing the cases that reflect the characteristics of long-channel and short-channel devices with that of an ideal linear input function implemented in software. Furthermore, by applying threshold voltage variation in the PGM state, the resulting degradation in recognition accuracy was analyzed. These results demonstrate that the short channel allows for the use of analog inputs with minimal accuracy drop compared to the ideal software-based model while also exhibiting strong robustness against threshold voltage variations, with an accuracy drop under 1% even under worst-case conditions. Moreover, by leveraging the high integration density and technological maturity of 3D-NAND flash arrays, the proposed approach demonstrated strong potential for neuromorphic computing applications.

## Data Availability

The original contributions presented in the study are included in the article; further inquiries can be directed to the corresponding author/s.
